# Security Framework for Pervasive Healthcare Architectures Utilizing MPEG-21 IPMP Components

**DOI:** 10.1155/2009/461560

**Published:** 2008-12-31

**Authors:** Anastasios Fragopoulos, John Gialelis, Dimitrios Serpanos

**Affiliations:** ^1^Industrial Systems Institute, R.C Athena, Stadiou Street, Platani, 26504 Patras, Greece; ^2^Department of Electrical and Computer Engineering, University of Patras, 26500 Rio Patras, Greece

## Abstract

Nowadays in modern and ubiquitous computing environments, it is imperative more than ever the necessity for deployment of pervasive healthcare architectures into which the patient is the central point surrounded by different types of embedded and small computing devices, which measure sensitive physical indications, interacting with hospitals databases, allowing thus urgent medical response in occurrences of critical situations. Such environments must be developed satisfying the basic security requirements for real-time secure data communication, and protection of sensitive medical data and measurements, data integrity and confidentiality, and protection of the monitored patient's privacy. In this work, we argue that the MPEG-21 Intellectual Property Management and Protection (IPMP) components can be used in order to achieve protection of transmitted medical information and enhance patient's privacy, since there is selective and controlled access to medical data that sent toward the hospital's servers.

## 1. Introduction

In modern computing era, health is an important aspect for society since it involves every citizen. The current health care system is designed to reward medical doctors and medical institutions to treat people when they are sick, and not acting proactively thus preventing them from being sick. Recently, in many countries the health care system is subject to reform since the rapidly growing populations of elderly combined with the increasing cost of health care services impose several challenges on health care providers, insurance companies, hospitals, and even on patients. All these suggest that first of all healthcare needs a major shift toward a more proactive direction focusing on prevention and/or early detection of acute events and afterwards more scalable and more affordable solutions should be offered. Therefore, all the above-mentioned requirements have accentuated the need for pervasive and ubiquitous embedded e-Health environments, given that limited financial and human resources will be committed.

The modern backbone communication infrastructures, networks, and technologies like GSM, UMTS, WCDMA, WiMAX, Internet, Wireless, and Bluetooth protocols provide extraordinary high data rates, thus allowing cost-efficient and time-efficient remote delivering of medical data that have been collected from the portable, embedded devices that reside onto the end-users (monitored patients) toward remote Medical Servers, for further processing. Furthermore, the pervasiveness and ubiquitous character of modern user-centric nomadic environments, in which the user is the central point surrounded by different types of embedded and small computing devices, like, for example, electrocardiograph (ECG) and pulse, oxygen saturation, and blood pressure sensors, add extra requirements concerning security aspects such as the protection of sensitive medical data and measurements, data integrity and confidentiality, and protection of patients privacy. In such environments, it is imperative to design and deploy efficient and effective network architectures as well as a generic, if possible, communication interface targeted to connect the external networks with the “smart” individual-person (equipped with different types of “smart” embedded medical devices (EMDs)) satisfying the basic security requirements for real-time, secure data communication, and protection of sensitive medical data and measurements, data integrity and confidentiality, and protection of the monitored patient's privacy. The architectures and the interface must consider the limited resources of the interconnected embedded systems, especially in light of the significant resources required for implementing security, which, in general, are quite resource-hungry leading thus to significant technical problems.

MPEG-21 is a standard that defines mechanisms and tools as means of sharing digital rights, permissions, and restrictions for digital content from content creator to content consumer. It is an XML-based standard that is designed to communicate machine-readable license information in an ubiquitous, unambiguous, and secure manner between peer entities. Although MPEG-21 is a standard that is used for sharing multimedia digital content, under well-defined terms, we argue that it could be used for protection of medical data and user's privacy in a pervasive healthcare environment, assuming that the digital content is the medical data generated by the EMDs. The content creator is any “user” under monitoring and the “end-user” is anyone having access to a remote Medical Server located in a hospital either being supervisors, trained medical stuff, or doctors.

In our work, we propose a generic security framework based on the MPEG-21 standard adopted for wearable EMDs thus filling an existing gap which made these devices and the data acquired by them vulnerable to any kind of attacks. Although the MPEG-21 standard has been adopted for delivery of multimedia content, we argue that it can be used in our architecture in order to enhance users' privacy and to enhance security requirements that are applied in such environments. Moreover, since the medical measurements that taken from patients' EMDs can include different data types, besides raw data, like, for example, a snapshot of electrocardiograph data in a jpeg file, or a video file of electrocardiograph data, the MPEG-21 standard can help us in content delivery, with security enhancement toward different types of end-users. Our endeavor demonstrates that no general-purpose home server is required for processing medical data before sent toward end-users, that is, doctors, medical staff, thus making the proposed architecture a good candidate for portable devices in pervasive healthcare environments exposing limited resources.

We strongly believe that the outcome of this work contributes toward the establishment of a standard context-aware security environment in which parts of sensitive personal information can be viewed only by valid users even though many users can have access to them. The main characteristics of our approach, which directly affects medical safety and treatment efficacy, comprise standardization, minimalizm, and sophistication. Ultimately, affects reduction of total health care cost, thus leading to a better utilization of limited healthcare resources.

More specifically, Section 2 gives a presentation of the relevant work, while Section 3 analyzes the emerging of EMDs security requirements in combination with MPEG-21 standard. Section 4 describes our proposed architecture and Section 5 provides the conclusions and the future work projection.

## 2. Related Work

Gialelis et al. [1] propose a pervasive healthcare architecture into which a wearable health monitoring system is integrated into a broad telemedical infrastructure allowing high-risk cardiovascular patients to closely monitor changes in their critical vital signs and get experts feedback to help maintain optimal health status. Consistent with the major challenge to provide good quality and reliable health care services to an increasing number of people utilizing limited financial and human resources, they propose a person-based health care system which consists of wearable Commercial of-the-shelf nodes which are already used in the hospital environment, and they are capable of sensing and processing blood-oxygen, blood-pressure, ECGs, and other vital signs and can be seamlessly integrated into wireless personal area networks (WPANs) for ubiquitous real-time patient monitoring. Their architecture lacks safety, security, and privacy considerations, which may lead to serious breaches to architecture's and EMDs functionalities or to users' privacy.

Venkatasubramanian and Gupta [2] made a survey on security solutions for pervasive healthcare environments focusing on securing of data collected by EMDs, securing the communications between EMDs and investigation of mechanisms for controlling the access to medical data. They propose the use of cryptographic primitives, where measurements of physiological values are used for cryptographic keys, eliminating thus the necessity for key distribution, for securing data, and for the establishment of secure communications between two entities. Concerning the access control to medical data, they survey methods that are based on role-based access control (RBAC), extending it for usage in pervasive healthcare environments.

As far as we know, the only proposal for usage of MPEG-21 as a mechanism to access control to medical records has been proposed by Brox [3]. The author links patients records into MPEG-21 digital items and attempts to find access control mechanisms based on MPEG-21 standard. In this work, the author does not provide a clear architecture that implements the usage of MPEG-21 and also he does not use the MPEG-21 Intellectual Property Management and Protection (IPMP) components for the protection of medical records, but he mentions its use as a future and open research issue.

## 3. Security Requirements—Threat Model

The primary function of a pervasive healthcare architecture is to collect, to process, and to store medical data from different types of EMDs, which are located on the patient, locally into general-purpose computing devices—for further handling or to remote-located servers. In order to identify the security mechanisms that should be deployed into the proposed architecture for pervasive healthcare with the utilization of EMDs, we must identify and classify (i) possible attackers and malicious users of the aforementioned environment; (ii) safety, security, and privacy requirements.

Halperin et al. [4] provide a short classification of possible malicious users and adversaries in a pervasive healthcare environment, identifying

- *active adversaries* which are malicious users that have explicit interference and physical access to an EMD (i.e., launching probing attacks, tampering attacks),
- *passive adversaries* which are malicious users that have implicit interference with an EMD, through eavesdropping the communication link between an EMD and another system or through side-channel attacks,
- *insiders* which comprise the most probable adversaries in such environments. Such attackers include healthcare personnel, nurses, doctors, IT professionals, and even the patients themselves.

Security is a general requirement in modern computing environments, but in pervasive healthcare systems security is an imperative requirement because those systems handle very sensitive data like medical data and electronic personal records (EPRs). In order to identify the security aspects and requirements in such environments, we have to identify the threat model; some of the most significant threats for pervasive healthcare environments are (a) *nonauthorized access to patient's medical data*, that is, a nurse and a doctor must have different authorities and access control to medical data; (b) *intentional alteration of medical data*, thus leading to incorrect diagnosis and patient's treatment; (c) *disclosure of medical data to third parties* (e.g., to insurance companies or any third parties that may use such records to gain profit), aiming to increase their revenue. In their work, Halperin et al. [4], also, describe a generic framework for analysis, design, and evaluation of security and privacy issues in implantable medical devices (IMDs). Although our architecture does not contain any type of IMDs, a subset of the security and safety goals that they describe in their work applies to us, also. They identify two directions, one concerning safety and utilization goals and the other concerning security and privacy goals, and furthermore they are trying to find tradeoffs and tensions between those directions. Safety and utility goals are traditional requirements in pervasive healthcare systems, involving some security aspects. Such goals are (a) *accessibility to medical data*, that is, ensuring that only appropriate entities must have access to EMDs and their medical data; (b) *accuracy of measurements and data*, that is, all data and measurements taken from the EMD must be accurate; (c) *traceability and identification of EMDs*, that is, an EMD must have mechanisms that allow it to make its presence clear to authorized entities, whenever it is necessary; (d) *maintenance and reconfigurability*, that is, authorized personnel may alter EMDs configuration, locally or remotely; finally, (e) *resources efficiency*, that is, extend EMDs battery life, through minimization of power consumption. Besides safety and utility goals, due to their nature, the modern pervasive healthcare environments have also to fulfill some security- and privacy-related goals and requirements. Those goals are not different from traditional security requirements of computing systems that basically rely on the attainment of authentication and authorization, data confidentiality, data integrity, availability and protection of users' privacy, and have to be extended and possibly reviewed in the context of pervasive healthcare environments. *Authentication.* Refers to methods and mechanisms which allow to an entity to prove to a remote end its identity, that is, in a transaction between two end-users over a possibly unsafe communication network, there must be mechanisms that assure that each part can be authenticated by a remote end. *Authorization*, Refers to access control mechanisms and to the ability of an entity to access shared resources. Two basic subcategories of authorization can be distinguished (a) *personal authorization*, that is, specific people or groups of people may access patients' data and perform specific action over it; (b) *role-based authorization*, that is, a person may have access to medical data based on a specific role that he has, for example, a doctor, a nurse, a caregiver. *Data integrity.* Mechanisms ensure that when there is an interchange of data between two peer entities, the received data and the original ones are the same, and that no intermediate alteration has occurred, for example, through interference of an eavesdropper. In a typical pervasive healthcare system, various messages and data are interchanged between different participating entities, so the integrity of the transmitted messages and data is a basic requirement. *Data confidentiality*, It assures that stored or transmitted data are well protected from possible disclosure. A mean to achieve data confidentiality is through cryptographic mechanisms. *Availability.* It is a security requirement which implies that a malicious user may not be able to passively or actively perform a denial-of-service attack to an EMD, for example, battery attack, memory overflow, jam the communication interface [5], thus preventing it from operating normal and smoothly. *Privacy*, It can be defined as an entity's ability to control how, when, and to what extent personal information about the entity will be communicated to third parties [6]. Anderson [7] defines privacy as the secrecy for the benefit of an individual entity, where secrecy refers to generic mechanisms that do not allow unauthorized usage and access of data and resources. Extending privacy in the context of pervasive healthcare environments, we refer to (i) *EMD-existence and EMD-type privacy*, that is, an EMD should not make its presence and its type clear to an unauthorized party, since the user (patient) might not want to know nonauthorized users what type of EMD they carry with them and what type of functions it serves.

Our proposed architecture addresses authentication and authorization issues, since each user has a license that defines what he/she has access to. Moreover, data confidentiality is achieved through cryptographic mechanisms, that is, sensitive data are encrypted and data integrity is achieved transparently by the doctor's devices that have access to the data. Finally, the utilization of a DRM mechanism through the MPEG-21 framework helps in the protection of user's privacy, that is, different groups of users have access to different types of medical data.

### 3.1. MPEG-21 Standard

**Figure 1:**
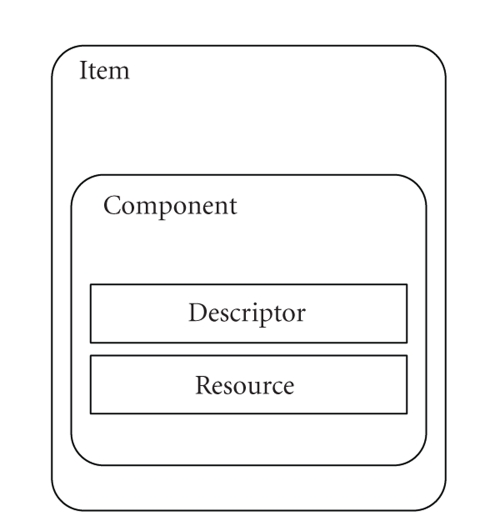
A schematic structure of an MPEG-21 digital item.

MPEG-21 is a new standard which has been proposed for primary use in the context of multimedia world, allowing the seamless, transparent, and universal delivery of multimedia content to the end-user, thus solving any interoperability issues. The core notion into the standard is the digital item (DI) that represents the digital asset (e.g., a movie, an audio track, or other data), and which contains the digital content and other related metadata (e.g., creator's details, usage rules and terms, licenses, security related information); the DI hierarchy is represented in Digital Item Declaration Language (DIDL) which is an XML-based document [8]. In the digital item hierarchy, see Figure 1, we identify (i) *resources* which are the individual multimedia data, that is, a picture, audio or video data, or any other data; (ii) *components* which are collection of resources with their descriptors. A component by itself is not an item, but the components are the basic building blocks of items, and (iii) *descriptors* which are metadata that accompany a resource, containing information that concerns all or part of the specific resource. Also, a *container* is a structure which comprises of items and containers, forming thus logical packages for interexchanges between entities.

### 3.2. MPEG-21 IPMP

**Figure 2:**
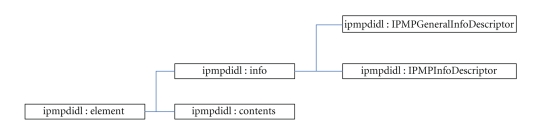
Structure of an IPMP DIDL element.

The security problems may arise from the fact that the digital item's description, that is, its structure, contents, attributes, and metadata, is a clear XML document and it is easily visible to anyone and vulnerable to nonauthorized usage. Due to that fact, the MPEG-21 includes a part named Intellectual Property Management and Protection (IPMP) which provide mechanisms for protection of digital item. More specifically, MPEG-21 IPMP in conjunction with the MPEG-REL Rights Expression Language (REL) provide a framework that enables all users in the digital contents delivery chain to express their rights and interests in digital items and to have the assurance that those rights and interests will be persistently and reliably managed and protected across a wide range of networks and devices [9]. The core notion in MPEG-21 IPMP is related with the IPMP tools that are used to protect the digital item. Those tools are not predescribed by the standard, but each user, vendor, and so forth, may define and implement his own set of tools which perform basic security functions like encryption/decryption algorithms, authentication and data integrity mechanisms, watermarking, and fingerprinting. With the use of MPEG-21 IPMP components, we may protect the whole DI or a part of it through the encapsulation of the original DIDL elements that we want to protect, with additional information (IPMP Info) that refer to mechanisms and tools for the protection of the original elements. MPEG-21 IPMP defines a new set of IPMP DIDL elements which have the same role and semantics as an element defined in DIDL. The structure of an IPMP DIDL element can be seen in Figure 2.

**Figure 3:**
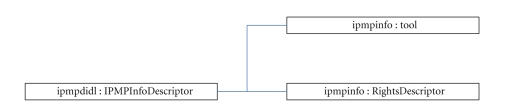
Schematic view of the structure of the *ipmpdidl : IPMPInfoDescriptor* element.

The *ipmpdidl : info* element contains information about protection and usage rules of the digital content, which may be categorized to (i) information about protection of the whole digital item, which is included in the child element *ipmpdidl : IPMPGeneralInfoDescriptor* and (ii) information about protection of a certain part of the digital item's hierarchy, which is included in the child element *ipmpdidl : IPMPInfoDescriptor*, see Figure 2. Both prementioned child elements have two purposes of existence (a) to describe the tools that are used for digital items protection, and (b) to provide a set of licenses that accompany the content and define its usage rules. More specifically, the *ipmpdidl : IPMPInfoDescriptor* element contains the following child elements (see Figure 3): (a) the *ipmpinfo : Tool* child element which is used to specify a tool (or tools) that protect the specified part of the DI's hierarchy. Each tool has a unique ID and may be either referenced, if it is located in a remote IPMP tools server or it may be included in line to the digital item (in the latter case, the base-64 encoded version of the tool is included); (b) the *ipmpinfo : RightsDescriptor* child element which contains the licenses that govern the usage of contents. Part 5 of MPEG-21 standard [10] defines the MPEG Rights Expression Language (REL) which a language that is used to create licenses that express usage rules and rights set by the creators of the digital items, regarding actions over them. Moreover, the license can be used to convey some other sensitive data, like, for example, decryption keys for the encrypted (protected) contents of the digital item. The MPEG-REL defines the *encryptedLicense* element which contains the license's contents in an encrypted form and it is used when the issuer wants to keep the content of the license confidential. When decrypted, the original license's contents are unveiled.

The child element *ipmpdidl : contents* contains the protected digital asset itself which has been protected with a set of tools (e.g., cryptographic tools, watermarking, hashing, digital signatures) that have been described in the child element *ipmpdidl : info*.

## 4. Proposed Architecture

**Figure 4:**
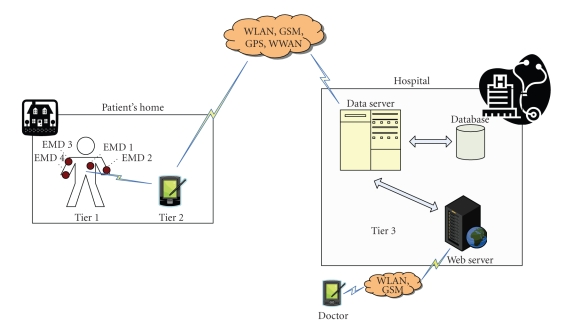
A high-level view of the proposed architecture.

In the high-level view of the proposed architecture as depicted in Figure 4, we can identify the user at home, equipped with different portable EMDs that transmit their measurements into a PDA which aggregates them for temporal storage and further processing (creation of encapsulated MPEG-21 IPMP-protected metadata file). Then, the generated file is sent to a data server which is located at the patient's hospital and it is stored to a database. If it is necessary, the patient's doctor may extract data from that file remotely through his PDA device, acting and informing accordingly the patient.

More specifically, Tier 1 encompasses a number of portable EMDs equipped with the corresponding sensors, easy to handle and maintain, which are integrated into a wireless wearable personal area network (WWPAN). Each of them can acquire, sample, and process one or more physiological parameters. More specifically, a small (5 cm × 5 cm) portable electrocardiogram device is utilized for the heart activity monitoring by providing a full 12-lead ECG by collecting signals through ten sensors and electrodes with fixed position. The ten input channels (VLA, VRA, VLL, VRL, V1-V6) are sampled at 500 Hz with a 10-bit resolution analog to digital (A/D) converter. A small (watch size) portable wrist (and arm if chosen) blood pressure device is utilized for monitoring patient's blood pressure upon request. Oxygen saturation is monitored through a small (2 cm × 2 cm) portable device which its sensor fits to one of patient's figures. Pulse rate is also measured either from the portable wrist or the ECG device.

Tier 2 encompasses a personal server application running on a personal digital assistant, a cellular phone, a laptop, or a home PC. The personal server undertakes a number of tasks such as a transparent interface to the WWPAN (portable devices), an interface to the user and an interface to Tier 3 (Medical Server & Thin Client). The interface to the WWPAN comprises network configuration and management tasks. The network configuration task, among other functions, supports device and sensor registration and initialization, customization, and network communication setup. After the network is configured, the personal server application is initiated in order to manage the network, that is, channel and time synchronization, data acquisition, data processing, data fusion, and so forth. Furthermore, upon the availability of a communication channel, the personal server establishes a secure link toward Tier 3 and transmits reports or files which are being further processed and integrated in the patient's health record. In case the availability of a communication channel is not possible, the personal server stores the data locally until the channel becomes available.

Tier 3 comprises the Medical Server which, actually, encompasses other servers such as emergency server, healthcare server, medical records server, mail server, and the “thin-client” used for remote accessing of Medical Server applications utilizing a “visualization” applet under secure link.

The Medical Server runs a large diversity of applications comprising setting up communication channel to personal servers, collecting users' reports, integrating data into patients' medical records data bases, processing medical records upon authorized doctors and/or specialized personnel demand, processing patients messages and other critical applications.

**Figure 5:**
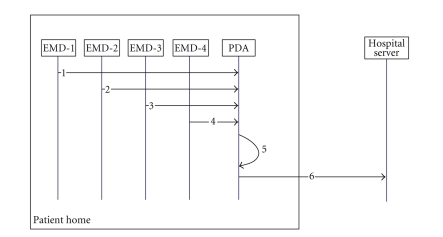
A sequence diagram of flow of medical data measurements from patient's home toward the data server which is located at the patient's hospital.

As soon as the medical data measurements are collected by the EMDs, they are sent toward the PDA (Tier 2) which is responsible for the creation of the MPEG-21-protected container that contains the medical measurements with appropriate usage licenses, as it can be seen in Figure 5.

In steps 1–4, medical data measurements from different user-located EMDs (ECG, blood pressure and oxygen saturation, and pulse rate) are taken and sent toward the PDA, which in turn, aggregates them and creates an initial packaged data file that contains the previous data measurements. Afterwards, the packaged file is amended to further processing in order to be “encoded” according to MPEG-21 terms, with appropriate metadata, protection information, and patient's instructions (relevant usage licenses) (step 5). Then, the new MPEG-21-based packaged file is sent to the hospital's servers for further handling, that is, storage in data servers, and informing physicians and doctors about patient's situation (step 6).

**Figure 6:**
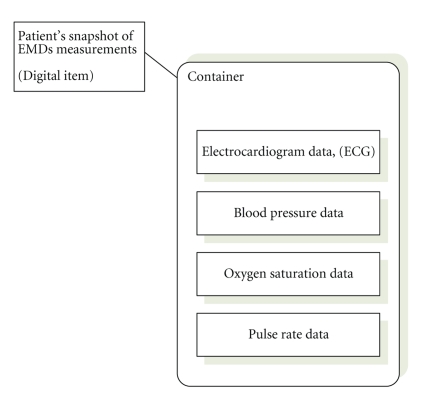
Abstract view of MPEG-21 digital item that presents a snapshot of EMDs measurements for a specific patient.

**Figure 7:**
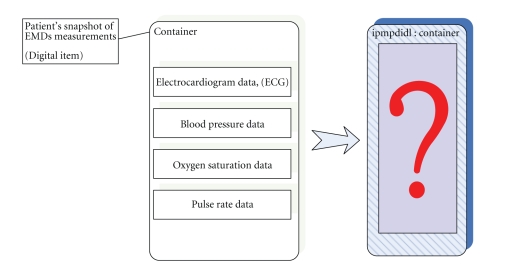
High-level view of encapsulation of a digital item using IPMP framework.

In the following figures, we depict an abstract view of an MPEG-21 digital item that contains EMDs measurements, Figure 6, and the schematic encapsulation of them into an IPMP container, see Figure 7. As we have stated before, the MPEG-21-based encapsulation of data protects it from unauthorized view and possible malicious usage.

In Section 4, we have emphasized that in such pervasive healthcare environments the protection of patient's privacy and medical data confidentiality are key issues. So when the MPEG-21-based packaged file comes to the hospital, its structure and its contents should not be fully accessible to nonappropriate users. We identify three groups of people that should have different access rights to the protected contents (i) the IT supervisors which are responsible for storing the received packaged files to databases, (b) the specialized medical personnel like, for example, the nurses, and (c) the doctors, who are responsible for the attendance of the patients. Each of these groups must have different access rights to protected digital contents. For example, the IT stuff may just see the patient's identification number, (patID), which is unique for each patient, in order to store the packaged file in data server and update patient's electronic record. On the other hand, a nurse or trained medical stuff must be able to see some details about the patient, for example, a part of patients' personal data like surname, name, and maybe an initial diagnosis, which may have automatically taken part at patient's home after the occurrences of data measurements, in order to inform the patient's doctor for further handling of the patient's situation. Finally, the patient's doctor may have full access to contents of MPEG-21-packaged files, that is, patients personal data, detailed medical data measurements taken by EMDs, time of measurements, and initial automated diagnosis. So the generated MPEG-21 container contains three different items; the first item is unprotected and contains a text data file with the patient's identification number, which can be used for storage of the file to a database for further handling, and some other data, like the time that the file was created at the user's PDA. The other two items are protected with the MPEG-21 IPMP components and are indented for use by the trained medical staff and the doctors, containing appropriate data. Each of these items contains a license of usage, in an encrypted form, which grants access to contents of file to specific user. The license also contains the key which has been used from the patient's PDA for the encryption of the medical data file. We assume that each patient and each of the doctors and trained medical staff have a prearranged set of public-private keys; those key pairs are assigned by the main hospital's server and can be revoked at any time since there is continuous communication between the hospital's server and the patient's (home) server. So the license for data usage can be encrypted using the public key of the entity with which it is related, for example, the doctor with whom the patient is related. In order to access the protected items' contents, the end-user must decrypt the license with his private key, retrieve the decryption key from the license, and decrypt the encrypted contents.

**Figure 8:**
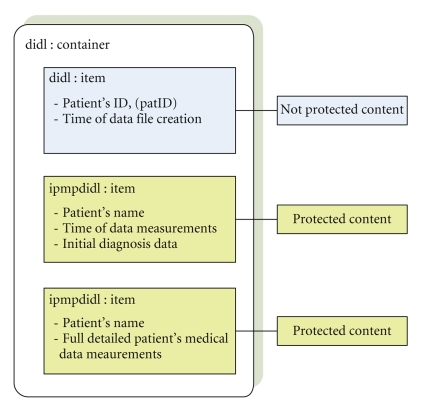
A schematic view of the encapsulated medical data into an MPEG-21 container. The first item is not protected, and it contains some data which can be used by the IT personnel in order to store the protected file into appropriate databases. The second item is protected, and it contains some part of personal data for the patient. Finally, the last item is also protected and includes the detailed medical data measurements. Each item is encrypted using different encryption keys, so each item can be seen only by users that possess the appropriate key for decryption.

Following, in Figure 8, we depict a schematic view of the encapsulated MPEG-21-based data file which is transmitted from patient's home server toward hospital data servers.

**Figure 9:**
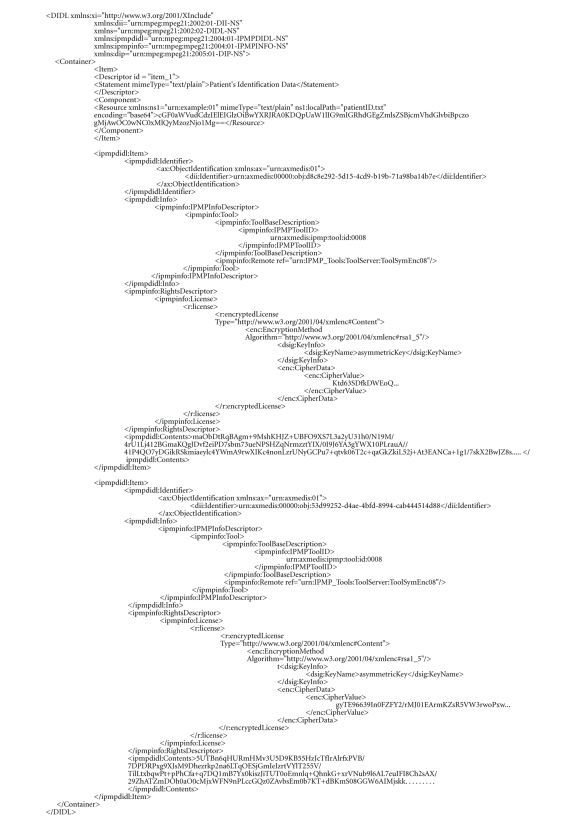
An example view of an MPEG-21 IPMP container which contains three items—one not protected and two protected with the use of a cryptographic tool that is remote referenced at the location *urn:IPMP_Tools:ToolServer:ToolSymEnc08.* Before the user decrypts the contents, he must decrypt the license that accompanies the content, which will provide to the user the decryption key and usage rules of the content. The license is binded to a specific user and it is included into the item encrypted using a public-key algorithm; in our implementation, we used RSA [11], and the license has been encrypted using the users' public key. For the creation of the aforementioned MPEG-21 IPMP-based file, we have used the AXMEDIS editor [12].

Finally, in Figure 9, we present an XML-view of the MPEG-21 IPMP-protected container that contains the three prementioned items. The contents of each item are protected (encrypted) with the use of a tool that implements a specific symmetric cryptographic algorithm, and it is remote referenced into the digital items' description. Even if one malicious user has access to the remote location of the tool, he needs some cryptographic sensitive information like, for example, a decryption key for decoding the protected contents; the key for the decryption is included into the encrypted license that accompanies each item, which furthermore defines the usage rules of the contents. Each item contains some information that identifies the user for whom this item is destined, that is, item 1 has to be processed by the IT stuff, item 2 has to be taken care by a nurse, and so forth. After the reception of each item, each relevant user initiates the procedure which will decrypt his/her license—with the use of his private key and then he/she will decrypt the protected contents—with the symmetric key that is embedded into his/her license, thus revealing the original packaged medical information.

## 5. Conclusions

We strongly believe that the outcome of this work contributes toward the establishment of a standard security environment in which parts of sensitive personal information can be viewed only by valid users even though many users can have access to them. The main characteristics of our approach, which directly affect medical safety and treatment efficacy, comprise standardization, minimalizm, and sophistication. Ultimately, affects reduction of total health care cost, thus leading to a better utilization of limited healthcare resources. Moreover, our endeavor demonstrates that no general-purpose home server is required for processing of medical data before sent toward end-users, that is, doctors, medical staff, thus making the proposed architecture a good candidate for portable devices in pervasive healthcare environments exposing limited resources. In our proposal, we use the MPEG-21 standards' IPMP components in order to enhance users' privacy and achieve security requirements that are applied in such environments. To our knowledge, there is no other security framework compliant with MPEG-21 that has been applied to protect such content, thus no comparison with other similar frameworks is provided. As a matter of fact, this aspect constitutes a future research activity. Furthermore, with respect to future work, we are planning to exploit some other elements of the MPEG-21 standard which, for example, would allow us to include (in line) into the IPMP-protected digital item the protection tools that are used to govern the protected item rather than remote referenced them, and also we could use some other elements of MPEG-21 IPMP that allow digitally authentication of the protection tools, that is, each tool carries a digital signature which validated each time the tool is used, before appliance of them to the protected contents. A lot of our research work is taken place investigating issues that are related with the security of our architecture under known attacks. Moreover, as the MPEG-21 is a quite new open standard that can be used for protection of sensitive data with the use of DRM, as a future work, we are investigating the identification of possible leaks and vulnerabilities of the standard, that may lead to attacks and a compromised system. We are also investigating issues that are related with the safe destruction of the medical data after their viewing; in that context, the use of trusted platform architectures [13] could be a direction that will lead to a solution. At this time, we assume that the hospital's server is a trusted one, and that the devices on which those data are viewed are also safe and trusted, and that there is no possibility for data storage or alteration.
